# Fragmented QRS: What Is The Meaning?

**DOI:** 10.1016/s0972-6292(16)30544-7

**Published:** 2012-09-01

**Authors:** Yutaka Take, Hiroshi Morita

**Affiliations:** 1 Department of Cardiovascular Medicine, Okayama University Graduate School of Medicine, Dentistry and Pharmaceutical Sciences, 700-8558, Okayama, Japan; 2 Department of Cardiovascular Medicine, Sakakibara Heart Institute of Okayama, 700-0823, Okayama, Japan; 3 Department of Cardiovascular Therapeutics, Okayama University Graduate School of Medicine, Dentistry and Pharmaceutical Sciences, 700-8558, Okayama, Japan

**Keywords:** fragmented QRS, cardiovascular implantable electronic device (CIED), myocardial scar, cardiac event

## Abstract

Fragmented QRS (fQRS) is a convenient marker of myocardial scar evaluated by 12-lead electrocardiogram (ECG) recording. fQRS is defined as additional spikes within the QRS complex. In patients with CAD, fQRS was associated with myocardial scar detected by single photon emission tomography and was a predictor of cardiac events. fQRS was also a predictor of mortality and arrhythmic events in patients with reduced left ventricular function. The usefulness of fQRS for detecting myocardial scar and for identifying high-risk patients has been expanded to various cardiac diseases, such as cardiac sarcoidosis, arrhythmogenic right ventricular cardiomyopathy, acute coronary syndrome, Brugada syndrome, and acquired long QT syndrome. fQRS can be applied to patients with wide QRS complexes and is associated with myocardial scar and prognosis. Myocardial scar detected by fQRS is associated with subsequent ventricular dysfunction and heart failure and is a substrate for reentrant ventricular tachyarrhythmias.

## Introduction

**Figure 1:**
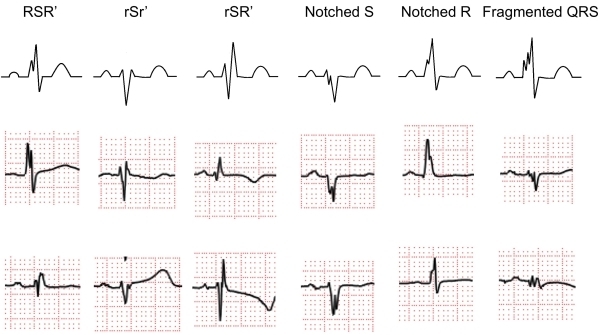
Classification of fragmented QRS (various RSR' patterns). Fragmented QRS was defined as an additional spike of QRS complexes without bundle branch block. Various RSR' patterns are present in the mid precordial lead or inferior lead.

It has been show in some studies that a subtle abnormality within the QRS complex can represent conduction disturbance and myocardial scar. A notch in the QRS complex in patients with left ventricular hypertrophy has been suggested to be a result of an intraventricular conduction defect [1]. Injured tissue around an infarct scar resulted in the RSR' pattern of the QRS complex [2]. However, the diagnostic and prognostic values of these subtle abnormalities within the QRS complex were not clarified in prior studies. In 2006, Das et al. proved fragmented QRS complex in patients with coronary artery disease (CAD) was associated with myocardial conduction block due to myocardial scar detected by myocardial single photon emission tomography (SPECT). fQRS was defined by an additional R wave (R') or notching within the QRS complex (Figure 1). fQRS improved identification of prior myocardial infarction in patients who are being evaluated for CAD. Since that report, the usefulness of fQRS for the diagnosis and prediction of prognosis has expanded to patients with ischemic and non-ischemic cardiomyopathy and patients with primary electrical diseases.

## Recording and definition of fQRS

ECG recording that is used to detect fQRS is not a specific setting and is the same as routine 12-lead ECG recording: high-pass filter: 0.05-20 Hz (usually 0.15 Hz), low-pass filter: 100-150 Hz, AC filter: 50 or 60 Hz, paper speed: 25-50 mm/sec (usually 25mm/sec) and voltage: 1mm/mV[3-5].

**Figure 2:**
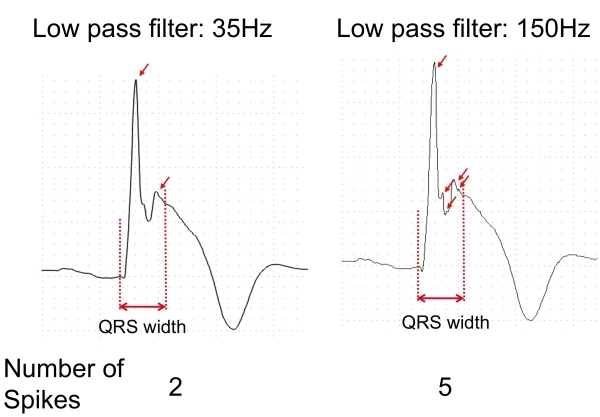
Effects of low-pass filter. ECG recording with a low-pass filter of 35Hz showed only 2 spikes within the QRS complex (left). Change of the cut-off frequency from 35 to 150 Hz unmasked 3 additional spikes within the QRS complex (right).

A low-pass filter is usually used to reduce electrical and musculature noises when recording the 12-lead ECG, but cut-off frequency of the low-pass filter influences detection of fQRS [6]. Figure 2 shows effects of the low-pass filter on the detection of the small spikes. ECG recording with a low-pass filter of 35 Hz showed only 2 spikes (R waves) within the QRS complex. Increasing the cutoff frequency of the low-pass filter from 35 to 150 Hz unmasked 3 additional spikes within the QRS complex (Figure 2).

Das et al. defined fQRS as the QRS complexes with the presence of an additional R wave (R') or notching in the nadir of the R wave or the S wave, or the presence of >1 R' (fragmentation) in 2 contiguous leads, corresponding to a major coronary territory. Typical bundle branch block (BBB) pattern (QRS ≥ 120 ms) and incomplete right BBB were excluded from the their original definition (Figure 1).

**Figure 3:**
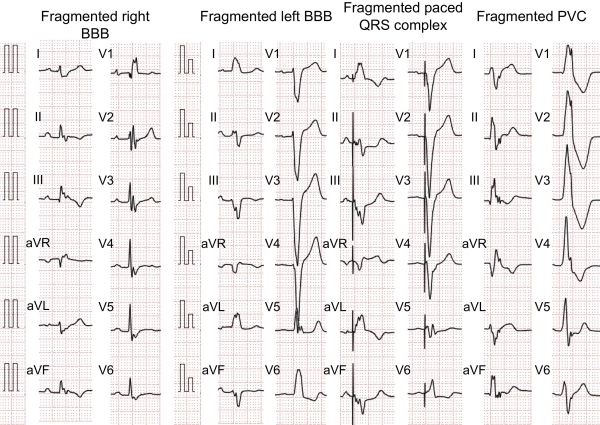
Fragmented wide QRS complex. Fragmented wide QRS complex in the bundle branch block (BBB), paced QRS complex and premature ventricular complex (PVC) have more than two notches.

fQRS on a 12-lead ECG was originally defined as narrow QRS complex duration (<120 ms) [3]. The QRS complexes of typical right/left BBB usually contained 1 additional R'. Is fQRS in a wide QRS complex a clinical indicator of myocardial scar? Das et al. added fQRS criteria in a wide QRS complex (≥ 120 ms): the QRS complex with >2 R' waves or notches in the R or S wave in a wide QRS complex of BBB, or paced QRS, or premature ventricular complexes (PVC) in 2 contiguous leads. If the QRS complex of PVC only has 2 notches in the R waves, they considered the QRS complex to be fQRS-positive when the notches were > 40 ms apart and present in 2 contiguous leads [4] (Figure 3).

When we reported fQRS in Brugada syndrome, we also defined fQRS in right BBB because patients with Brugada syndrome often had right BBB [6]. Most (64%) of the 80 control subjects with right BBB but without known heart disease and risk factors of atheroscrelosis (53 cases with complete right BBB and 27 cases with incomplete right BBB) had 2 to 3 spikes within the QRS complex in each of the right precordial leads (leads V1-V3) and a sum of 5.9 ± 1.0 (median: 6, range: 4-8) spikes in all V1-V3. We defined presence of fQRS in right BBB as 1) ≥4 spikes in one or 2) ≥8 spikes in all of the leads V1, V2 and V3. Two control subjects (2.5%) were regarded as having fQRS by this criterion. In this criterion, elderly subjects and subjects who had risk factors of atherosclerosis (diabetes, hypertension, etc) often had fQRS. This criterion focused on right precordial leads (V1-3) to identify conduction abnormality in the right ventricle and might not include abnormality of the left ventricle.

## Mechanism of fQRS

**Figure 4:**
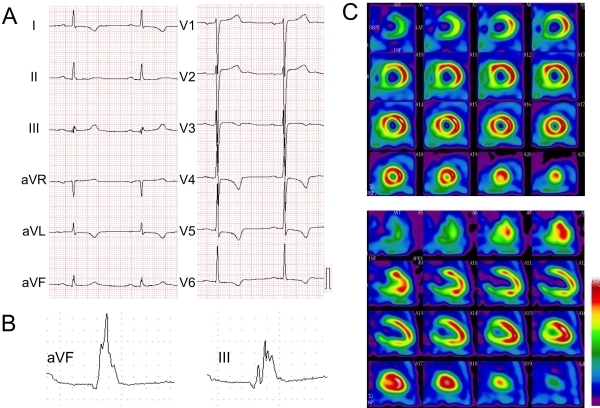
Fragmented QRS in a 64-year-old patient with old myocardial infarction. A) 12-lead ECG did not have an abnormal Q wave, B) multiple R waves were present in III and aVF leads, and C) nuclear imaging (99m Tc-TF) showed a fixed inferior myocardial perfusion defect.

fQRS can be caused by zigzag conduction around the scarred myocardium, resulting in multiple spikes within the QRS complex [3,4,6,7]. Reddy et al. showed that fQRS in left precordial leads in the absence of BBB indicated a sign of left ventricular aneurysm by left ventricular angiography [8]. Myocardial single photon emission tomography (SPECT) can identify regional perfusion abnormalities from a scar by a prior myocardial infarction. Studies in which the diagnostic values of Q wave and fQRS for a myocardial scar detected by SPECT were compared showed that fQRS was associated with significantly greater perfusion and functional abnormalities than was the Q wave [3,4] (Figure 4). The presence of fQRS in anterior leads (V1-5) predicts myocardial scar in the anterior myocardial segment or in the left anterior descending territory. The presence of fQRS in lateral leads (I, aVL, and V6) predicts myocardial scar in the lateral myocardial segment or left circumflex territory myocardial scar. The presence of fQRS in inferior leads (II, III, and aVF) predicts myocardial scar in the inferior myocardial segment or in the right coronary artery territory [3,4,9].

**Figure 5:**
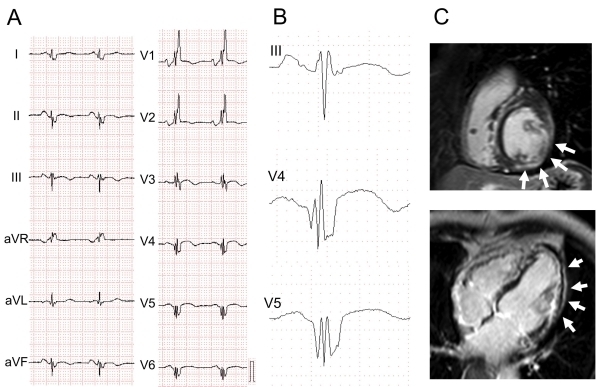
Fragmented QRS in a patient with dilated cardiomyopathy. ECG and images from a 74-year-old patient with left ventricular dysfunction (ejection fraction: 32%). The patient was diagnosed as having non-ischemic dilated cardiomyopathy. A) 12-lead ECG showed right bundle branch block, B) fQRS (various RSR' patterns) was present in left lateral and inferior leads, and C) delayed enhancement in Ga-MRI was present in the inferolateral resion (white arrows).

Late gadolinium enhancement cardiac magnetic resonance (Ga-MRI) is another tool for identifying the myocardial fibrosis and dysfunction. Abnormal late enhancement of gadolinium in patients with cardiac dilated cardiomyopathy (DCM) [10,11] (Figure 5) or cardiac sarcoidosis [12] or repaired tetralogy of Fallot [13] was associated with the existence of fQRS. Myocardial scar and conduction disturbance result in dyssynchrony in left ventricular systolic function. Fragmented QRS was associated with intra-ventricular systolic dyssynchrony in patients with narrow QRS [10,11]. Patients with fQRS might benefit from cardiac resynchronization therapy.

We studied the mechanism underlying fQRS in an experimental model of Brugada syndrome [6]. We created simulated conduction disturbance in the canine right ventricular tissues having drug-induced Brugada syndrome. Local epicardial delay caused multiple spikes at the late phase of the QRS complex resembling fQRS in the 12-lead ECG.

## Pathophysiology of fQRS

fQRS represents myocardial scar and will be associated with ventricular dysfunction and occurrence of congestive heart failure. In CAD, fQRS represents prior occurrence of myocardial infarction and will have a risk of subsequent occurrence of ischemic events. Indeed, Das et al. demonstrated that fQRS is an independent predictor of cardiac events in patients with CAD [5].

Myocardial scar is also a substrate for reentrant ventricular tachyarrhythmia. A signal averaged electrocardiogram (SAECG) reveals the presence of late potential that indicates low-amplitude high-frequency potentials outside the terminal QRS complex. Abnormal late potential represents a slow conduction zone with damaged myocardium around the fibrosis of healed myocardial infarction [14]. The presence of late potential has been used for risk stratification of sudden cardiac death or lethal arrhythmic events [15]. As well as SAECG, fQRS also can reflect intra-cardiac conduction abnormality and will represent a substrate for ventricular arrhythmia [3,6]. There has been only one study in which the correlation between fQRS and late potential detected by SAECG was investigated. That study demonstrated that the existence of fQRS appeared independently from the existence of late potential in patients with Brugada syndrome [6], but it is still unknown whether there is a correlation between fQRS and late potential in other diseases such as CAD and various cardiomyopathies.

## Diagnosis of myocardial scar in coronary artery disease

Results of the first two studies on fQRS in CAD were reported in 2006 [3,8]. Das et al. reported the definition of fQRS in a 12-lead ECG [3]. They used myocardial SPECT for detecting the scar area of the left ventricle and compared the clinical significance of fQRS and Q wave for diagnosis of regional perfusion abnormality in patients with CAD and a narrow QRS complex (Figure 4). The Q wave was present in 14.8% of the patients and fQRS was present in 34.9% of the patients. fQRS had higher sensitivity (85.6%) and negative predictive value (92.7%) for detecting the myocardial scar than did the Q wave, whereas the Q wave had higher specificity (99.2%) than that of fQRS (89%). The combination of Q wave and fQRS improved the sensitivity (91.4%), specificity (89%), and negative predictable value (92.4%) for detection of scared myocardium. They also reported that fQRS had a high negative predicative value for the detection of left ventricular aneurysm [8]. In patients with chronic total occlusion without prior myocardial infarction, existence of fragmented QRS was associated with poorly grown collateral coronary circulation.[16] Regional fQRS patterns show the presence of a focal regional myocardial scar [17], and laterality of fQRS represents a lesion in the territory of the coronary artery [3,18]. Precordial small Q wave and/or fragmentation in leads V2 and V3 could predict the presence of coronary artery stenosis in the left anterior descending branch [19]. A regional scar results in localized systolic dysfunction, and fQRS was associated with local LV dysfunction detected by 2D strain imaging in CAD patients with preserved ejection fraction (EF) [20].

fQRS also had diagnostic value for detection of a myocardial scar in patients with CAD and a wide QRS complex. In the presence of wide QRS duration, fragmented wide QRS (f-wQRS) was present in 47.2% of patients and had good sensitivity and specificity for detection of myocardial scar. fQRS in a wide QRS complex had good sensitivity (86.8%), specificity (92.5%), positive predictive value (92.0%), and negative predictive value (87.5%) for detecting scarred myocardium [4].

Although some studies have shown the clinical significance of fQRS for detection of myocardial scar in patients with CAD, one study failed to show significance of fQRS for detection of myocardial scar [21]. The reason for the different result from previous studies is unclear, and the authors stated that larger study is required to confirm the diagnostic value of fQRS.

## Prognosis of coronary artery disease

Since fQRS represents myocardial scar, fQRS may be associated with heart failure and ventricular tachyarrhythmia. Some studies have shown a relationship between existence of fQRS in patients with CAD and prognosis. In patients having narrow QRS complexes (< 120 ms), all-cause mortality (34.1% vs. 25.9%) and cardiac event rate (49.5% vs. 27.6%) were higher in patients with fQRS than in patients without fQRS [5]. Multivariate analysis revealed that existence of fQRS was an independent predictor for cardiac events (hazard ratio, HR: 1.62) as well as EF (HR: 1.40) and perfusion disturbance detected by SPECT (HR: 1.32). A study in which fQRS was evaluated by a body surface mapping system also showed that fQRS was an independent predictor for cardiac death (HR: 8.7) and hospitalization due to heart failure (HR: 3.8) in patients with prior myocardial infarction [22]. Torigoe et al. evaluated fQRS by 12-lead ECG and showed that the number of the leads with fQRS was a predictor for cardiac death and hospitalization for heart failure in patients with prior myocardial infarction (HR: 1.33) [23]. Although prior criteria for positive fQRS included the presence of fQRS in 2 or more contiguous leads, they showed that the presence of fQRS in 3 or more leads was the most useful for distinguishing between patients with and without risk for cardiac death or hospitalization. An increase in the number of leads with fQRS would represent a wide scar area, which would result in an adverse outcome. fQRS also predicted intra- and post-operative hemodynamic instabilities and adverse cardiovascular events in patients undergoing coronary artery bypass graft surgery [24].

Patients with organic heart disease often have right or left BBB, and wide QRS complexes are associated with adverse prognosis for the patients. For example, the HERO-2 trial [25] showed that mortality of patients who had left/right BBB with anterior AMI or patients who had right BBB with inferior AMI was higher than that of patients with AMI but no sign of BBB. Although original criteria for fQRS excluded wide QRS complex ≥ 120 ms [3], fQRS with BBB could also be considered as a predictor of mortality. Das et al. found that fQRS was associated with poor prognosis in patients with CAD and wide QRS complex [4]. Mortality of patients with f-wQRS was significantly higher than that of patients without f-wQRS, and subgroups of f-wQRS (fragmented left BBB, fragmented PVC and fragmented paced QRS) were also associated with short time to death compared to patients without f-wQRS. Multivariate analysis showed that reduced EF (≤ 35%, relative risk, RR: 2.27), age (RR: 1.06), and f-wQRS (RR: 1.41) were independently associated with mortality.

## Acute coronary syndrome

In acute coronary syndrome, fQRS appeared within 48 hours (especially within 24 hours) from the onset of symptoms and persisted thereafter [18]. fQRS on 12-lead ECG developed in 55% of patients with ST elevation myocardial infarction (STEMI) and in 50% of patients with non ST elevation myocardial infarction (NSTEMI), but in only 3.7% of patients with unstable angina pectoris (UAP). A new Q wave occurred in 44% of patients with STEMI, 23% of patients with NSTEMI and 0.4% of patients with UAP. Although the sensitivities of fQRS for STEMI and NSTEMI were 55% and 50% respectively, the specificity of fQRS for AMI was 96%. All-cause mortality of patients with fQRS was higher than that of patients without fQRS. In multivariate analysis, fQRS was an independent predictor for all-cause mortality (HR: 1.68) and was superior to Q wave (HR: 1.47). Ari et al. [26] found that appearance of fQRS after 48 hours from the onset of AMI was a predictor for cardiac events (death, AMI, revascularization) in patients with STEMI who had undergone primary PCI. Pietrasik et al. [27] showed that patients with fQRS and resolved Q wave 2 months after AMI had a more than twofold higher risk of recurrent events than did those without fQRS and persistent Q waves. However, enrollment for this study was performed in 1988-1991, and the therapeutic strategy would be different from the recent one and would be a limitation of that study.

## Dilated cardiomyopathy

The correlation between fQRS and ischemic/non-ischemic cardiomyopathy with reduced LV function was investigated in some studies. fQRS was present in 23-75% [7,10,11,28-30] of the patients with dilated cardiomyopathy (DCM) and narrow QRS complexes and was common in patients with ischemic cardiomyopathy [28]. fQRS was associated with intraventricular dyssynchrony in patients with non-ischemic DCM and would be useful for identifying patients who benefit from cardiac resynchronization therapy [10,11].

A wide QRS complex was observed in 14-47% of patients with heart failure. A wide QRS complex, especially left BBB, is associated with more advanced myocardial injury, worse left ventricular function and higher mortality than those in the case of a narrow QRS complex [31] (Figure 5). Two studies showed that fQRS as well as wide QRS complex was associated with worse prognosis in patients with DCM [7,30]. In patients with non-ischemic DCM (EF ≤ 40%), fQRS was a strong predictor of mortality and arrhythmic events, and event-free survival in patients with fQRS or wide QRS complex was significantly decreased than patients without fQRS and wide QRS complex. [30]. fQRS was an independent predictor of lethal arrhythmic events (HR: 7.62) (implantable cardioverter-defibrillator (ICD) shock or antitachycardia pacing) in patients with ischemic or nonischemic DCM who had received an ICD for primary or secondary prophylaxis, but it could not predict death in that population [7]. If the subjects had been limited to DCM patients (both ischemic and nonischemic) with primary prevention by an ICD, the usefulness of fQRS for predicting arrhythmic events might have been lost. Two studies showed that fQRS was not associated with a higher risk of both arrhythmic events and mortality in patients with an ICD for primary prevention [28,29]. fQRS also failed to predict infarct size in patients with severe LV dysfunction (EF 27%) and wide QRS complex [28].

## Cardiac sarcoidosis

**Figure 6:**
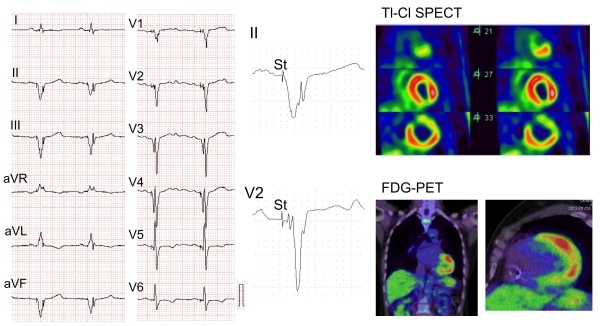
Fragmented QRS in a 52-year-old patient with cardiac sarcoidosis. A) 12-lead ECG showed a wide QRS complex by right ventricular apex pacing, B) fQRS was present in inferior and mid-precordial leads, C) Thalium-201 myocardial perfusion imaging showed an anterolateral-infero perfusion defect. 18F-fluoro-2-deoxyglucose positron emission tomography (18F-FDG-PET) showed FDG accumulation at the same site of the perfusion defect.

Sarcoidosis is a granulomatous disease that affects systemic organs. Cardiac involvement of sarcoidosis induces atrio-ventricular block, right BBB, ventricular tachyarrhythmia, and heart failure and is associated with adverse outcomes in the patients. The presence of fQRS on a 12-lead ECG in patients with sarcoidosis was associated with cardiac involvement detected by late enhancement on Ga-MRI [12]. Forty-six percent of patients with pulmonary sarcoidosis also had cardiac sarcoidosis [32]. BBB (right BBB: 23.1%, left BBB: 3.8%) and QRS widening were frequently observed in patients with cardiac sarcoidosis. Seventy-five percent of patients with cardiac sarcoidosis had fQRS, whereas only 34% of patients without cardiac sarcoidosis had fQRS. The presence of fQRS or BBB was related to cardiac sarcoidosis among patients with pulmonary sarcoidosis (Figure 6).

## Arrhythmogenic right ventricular cardiomyopathy

Arrhythmogenic right ventricular cardiomyopathy (ARVC) is characterized pathologically by fibrofatty replacement of the right ventricular myocardium and ventricular tachycardia [33]. Epsilon wave is a delayed potential at the terminal phase of the QRS complex and represents right ventricular (RV) delayed conduction. Epsilon wave is specific for ARVC, but the incidence of epsilon wave is not high in patients with ARVC. fQRS was found in 85% of patients with ARVC, whereas epsilon wave appeared in only 23% of the patients, [34] and highly amplified ECG increased detection of the epsilon potential to 77% of the patients. The number of leads with fQRS in patients with ARVC was associated with a severe form of the disease including LV involvement.

## Electrical diseases

Brugada syndrome is characterized by cove-type ST elevation in right precordial leads and episodes of ventricular tachyarrhythmia. Experimental studies have shown that ST elevation and arrhythmia result from right ventricular repolarization abnormality. Some clinical studies have also shown the existence of depolarization abnormality and it was also associated with high-risk patients [35]. We evaluated the significance of f-QRS in patients with Brugada syndrome [6]. Patients with Brugada syndrome often had fQRS and it was more frequently observed in the VF group (incidence of fQRS: VF 85%, syncope 50%, and asymptomatic 34%, P<0.01). fQRS was not associated with the existence of late potential recorded by a SAECG. Patients who had fQRS often experienced recurrent syncope due to VF within 4 years of the first syncope or ventricular fibrillation episodes. Recently, the PRELUDE study has shown that fQRS was useful for identify candidates for a prophylactic ICD implantation in patients with Brugada syndrome (HR: 4.902) as well as spontaneous type 1 ECG, history of syncope, and short ventricular refractory periods [36].

Acquired long QT syndrome (ALQTS) is a disease due to secondary repolarization abnormality. Repolarization abnormality, such as prolongation of QT interval and prolongation of the peak to end of T wave represented transmural or intraventricular dispersion of repolarization, and was associated with occurrence of tosades de pointes [37]. We also found that fQRS was present in a large percentage of ALQTS patients with syncope/torsades de pointes (81 %) [38]. Although onset of torsades de pointes is triggered activity due to early afterdepolarization, myocardial scar that appears as fQRS could be a substrate for subsequent reentrant arrhythmia. 

## Other conditions

In adult patients with repaired tetralogy of Fallot [13], fQRS predicted ventricular fibrosis detected by late Ga-MRI. Laterality of fQRS was associated with operative scar of the RV, and patients with fQRS had larger RV volume and lower RV EF. fQRS was closely associated with more extensive RV fibrosis and dysfunction.

fQRS was frequent in patients with mitral stenosis caused by rheumatic fever [39]. Rheumatic fever induces inflammation and degeneration of the cardiac valve and also injury of the myocardium. fQRS was associated with low EF, pulmonary hypertension, poor NYHA functional class, and decreased mitral valve area.

## Future directions and conclusion

**Figure 7:**
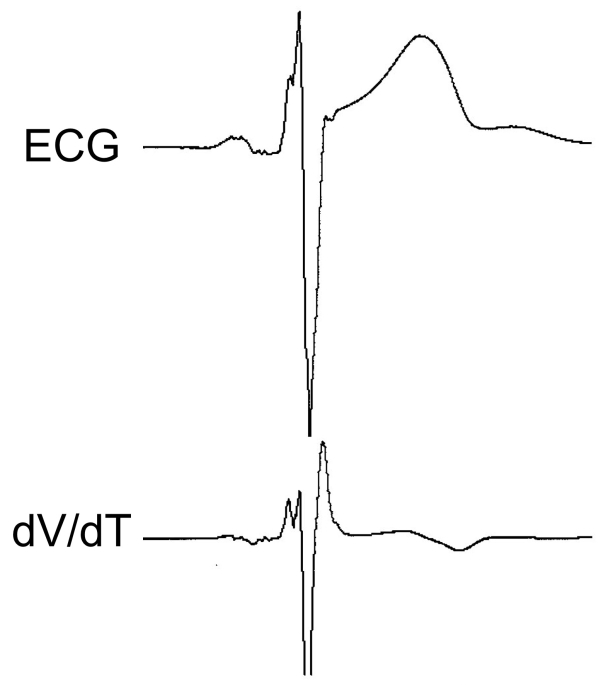
New analysis of fragmented QRS using the first derivation of voltage by time (dV/dT) of the QRS complex. Spikes and notches within the QRS complex were clearly represented as positive peaks in the dV/dT analysis. ECG and dV/dT of the QRS complex were recorded by FX-7524 of Fukuda Denshi Co. Ltd and analyzed by original software.

fQRS is a useful marker of myocardial scar and can predict cardiac events and mortality in various heart diseases. However, some studies showed negative data of fQRS for diagnosis and prognosis of the diseases. For improvement of diagnosis and prediction of prognosis, qualitative analysis of fQRS might be required. Magnetoelectrocardiography is a possible method to evaluate qualitative analysis of fQRS, but it cannot be used as a routine examination [40]. We have recently developed a new method for analysis of fQRS using the first derivation of dV/dT of the QRS complex to evaluate fQRS conveniently (Figure 7). This method can identify an additional r' wave and notching observationally and will be useful for qualitative analysis of fQRS.
